# Treatment of Paralysis by Hypodermic Injections of Strychnine

**Published:** 1868-11

**Authors:** 


### TREATMENT OF PARALYSIS BY HYPODERMIC INJECTIONS OF STRYCHNINE.

M. Gonzalez Echeverria, M.D., in a paper read before the Connecticut Medical Society, May 28th, 1868, narrated several cases of paralysis treated by him, by hypodermic injections of strychnine, with remarks on infantile palsy.

These cases were selected by him as evidences of subcutaneous injections, proving by themselves an efficient means of treatment.

*Case 1.* A soldier, after being kept on picket duty all night, in water up to his knees, was admitted into the Central Park U. S. A. General Hospital, with paraplegia. The limbs were blue and cold, without sensibility, up to the knees. Lithates were abundant in the urine. The bowels and bladder were torpid. By the application of electricity to the limbs, and the internal use of tonics and strychnine, the paralyzed condition was slowly relieved. One-sixtieth of a grain of sulphate of strychnine was injected below the knee. The operation was repeated four or five times, at intervals of three days, and from the first injection, the patient, to his great joy, could walk without crutches. A feeling of warmth was noticed after the first puncture, with marked diaphoresis.

*Case 2.* A gentleman, on account of a sudden exposure to cold, while playing tenpins, became paralytic in the right leg. The patient, from the onset, was in constant agony from pain. Slight temporary relief was obtained by the use of blisters over the hip-joint, and the employment of narcotics. Soon the muscles of the thigh and leg began to waste away, and hyperæsthesia persisted to such an extent that the slightest touch of the skin would cause excruciating pain. Chlorides were found in excess in the urine.

After one-fiftieth of a grain of strychnine was injected into the thigh, the application of electricity was resorted to—as the patient stated that electricity had given him some relief previously. After the first puncture, more power and warmth were felt all over the limb. The pain subsided, and for the first time he enjoyed a night of uninterrupted rest. Diaphoresis followed, and the pupils dilated. More or less gurgling of the bowels seemed to be one of the earliest effects of the subcutaneous injection of strychnine. At intervals of four days, the same dose of strychnine was injected three times more. Power was restored to the paralyzed muscles, by a tonic regimen, with electricity; the hot and cold douche to the limb, and two more subcutaneous injections of one-fiftieth of a grain of strychnine.

*Cases 3 and 4.* These cases were of simultaneous paralysis in two children, brother and sister; the boy one and a half years old, the girl three. They were seized with symptoms of spinal meningitis, produced by sitting on the wet grass. The children were seen after the acute stage of the disease was passed. The girl had lost all power of both legs and right arm. The boy was only paraplegic. Hypodermic injections of strychnine were suggested, which being approved by the attending physicians, he injected five times—once every three days—one-fiftieth of a grain in the legs of the boy and over the lower part of the spine, who was able to walk, with a little unsteadiness, in the course of six weeks. Tonics, warm baths, electricity, and strychnine by the mouth, finished the cure. The girl did not progress as fast. She had fourteen injections of one-fiftieth of a grain of strychnine during three months. She could stand or walk, but became fatigued very easily, and the arm and hand recovered power to grasp more firmly. Electricity and the same treatment were kept up as with her brother, but she was not cured, though benefited considerably, on account of the absence of the parents from New York.

In these cases—as in other similar cases—fibrillar contractions of the muscles in the limbs, lasting for a minute or two, were noticed.

*Case 5.* A boy four years of age, affected with paraplegia, which had existed for two years, was treated by him. The paralysis followed a fever. The muscles of both legs were much wasted from the knee down to the feet, which were affected with talipes equinus. The child could only move about on his knees. When first seen he was suffering with bronchitis, following scarlatina.

Local applications of electricity to the palsied muscles were advised, with hypodermic injections of strychnine. The injection was practised in the anterior region of the limbs, one-sixtieth of a grain of strychnine being introduced in the tibialis anticus. Every third day, during four months, one-sixtieth of a grain was injected, also the daily application of electricity, for half an hour. The child recovered to such a degree that he could stand or walk, with the assistance of an orthopœdic apparatus.

Several more cases were described at length in his paper, but the five already mentioned show the importance of subcutaneous injections of strychnine in the treatment of paralysis.

The results observed by the author, and by the physicians quoted in his paper, "strongly indicate the cardinal part of the sympathetic in the pathogeny especially of infantile paralysis."

Strychnine administered by the mouth, or hypodermically, has an entirely different effect. By the former, the quantity may be increased and repeated unsuccessfully; while by the latter—in a smaller dose—the lost muscular power is restored.

As to the manner of performing the injections, his own language will explain: "Generally, I insert the trocar of the syringe into the paralyzed muscle, and draw part of it out to avoid throwing the solution directly into the bloodvessel. The injection should be practised very slowly; by having a solution with one-hundredth or a smaller fraction of a grain to a drop, the strychnine may be so diluted as to allow carrying its action at the same time into more than one of the palsied muscles. The general effects are more rapid and decided when the solution penetrates no deeper than the cellular tissue, or when the puncture is made along the spine."

